# A New Genetically Encoded Single-Chain Biosensor for Cdc42 Based on FRET, Useful for Live-Cell Imaging

**DOI:** 10.1371/journal.pone.0096469

**Published:** 2014-05-05

**Authors:** Samer Hanna, Veronika Miskolci, Dianne Cox, Louis Hodgson

**Affiliations:** 1 Department of Anatomy and Structural Biology, Albert Einstein College of Medicine of Yeshiva University, Bronx, New York, United States of America; 2 Gruss-Lipper Biophotonics Center, Albert Einstein College of Medicine of Yeshiva University, Bronx, New York, United States of America; National Cancer Institute, United States of America

## Abstract

Cdc42 is critical in a myriad of cellular morphogenic processes, requiring precisely regulated activation dynamics to affect specific cellular events. To facilitate direct observations of Cdc42 activation in live cells, we developed and validated a new biosensor of Cdc42 activation. The biosensor is genetically encoded, of single-chain design and capable of correctly localizing to membrane compartments as well as interacting with its upstream regulators including the guanine nucleotide dissociation inhibitor. We characterized this new biosensor in motile mouse embryonic fibroblasts and observed robust activation dynamics at leading edge protrusions, similar to those previously observed for endogenous Cdc42 using the organic dye-based biosensor system. We then extended our validations and observations of Cdc42 activity to macrophages, and show that this new biosensor is able to detect differential activation patterns during phagocytosis and cytokine stimulation. Furthermore, we observe for the first time, a highly transient and localized activation of Cdc42 during podosome formation in macrophages, which was previously hypothesized but never directly visualized.

## Introduction

Cdc42 is a member of the p21 Rho family of small GTPases that has been found to be implicated in a variety of signaling events and cellular functions [1]. Cdc42 regulates a myriad of downstream effectors including kinases such as p21 activated kinases (PAK), mixed-lineage kinases (MLK) and scaffolding proteins including Par6, Wiskott Aldrich Syndrome protein (WASp) and IQGAP [2]–[4]. Through these and many other downstream effectors, Cdc42 tightly regulates a variety of cellular processes including cell polarity, re-organization of the cytoskeleton, transcription, proliferation, adhesion, migration and membrane trafficking. Therefore, it is crucial that Cdc42 activity is tightly controlled to maintain normal cellular function, similar to that seen with other Rho GTPases that control many important signal transduction pathways. The activity of Rho GTPases is mainly regulated through nucleotide binding and subcellular localization [5]. Previously, a Cdc42 biosensor was developed to detect the activity of endogenous Cdc42 in living cells [6]. This MeroCBD biosensor system required *in vitro* production of the biosensor protein, solvatochromic organic-dye labeling chemistry, and microinjection of single cells, making the approach cumbersome to use for routine imaging purposes [7]. Single-chain, genetically encoded biosensors for Cdc42 based on the fluorescence resonance energy transfer (FRET) are also available [8], [9]. While vastly easier to implement due to the genetically encoded approach of these sensor systems, the design of these probes did not allow for the appropriate interaction with the direct upstream regulator of Rho GTPases, namely guanine nucleotide dissociation inhibitor (GDI) [8], [9]. Thus, these sensors do not fully reflect the regulatory cycle of GTPase activations in live cells.

Here, we report the development of a new, genetically encoded, single-chain biosensor for Cdc42 based on FRET. The biosensor incorporates the monomeric Cerulean (mCer) and monomeric Venus (mVen) fluorescent proteins as the donor/acceptor FRET pair. The key difference from the previous genetically encoded systems [8], [9], is that this biosensor was designed in a way to conserve the C-terminal hypervariable region and the prenylation motif of full-length endogenous Cdc42. This allows proper translocation of Cdc42 to the cell membrane upon activation, as well as, proper interaction with the upstream regulator GDI, maintaining normal shuttling between the cytoplasm and the membrane during its activity cycle [10]. As proof of principle, we have used this Cdc42 biosensor in mouse embryonic fibroblasts examining constitutive protrusion – retraction events and directly comparing these results to those using the MeroCBD biosensor [6] with morphodynamics analysis as a readout, useful for characterization of the Rho family GTPase activity at the leading edge [11]. We then extend our observations to a different cell type, namely macrophages, and show differential Cdc42 activation during phagocytosis, cytokine stimulation and podosome formation.

## Results and Discussion

**Figure 1:**
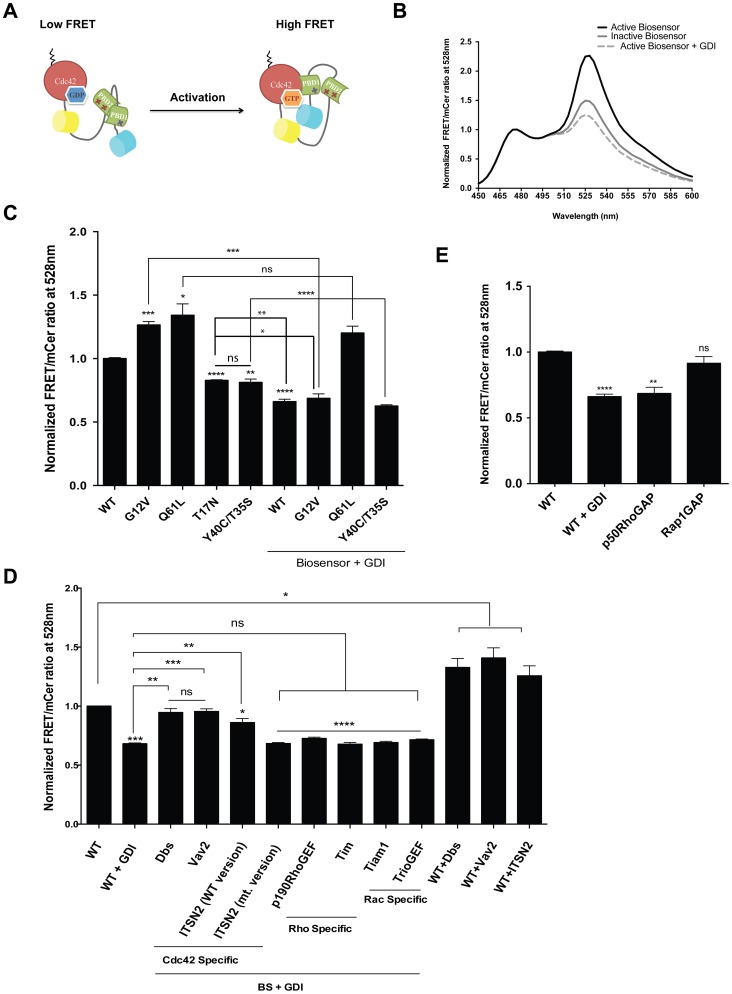
Cdc42 biosensor design and characterization. **A**) Diagram illustrating the design of the single-chain Cdc42 biosensor. H83D mutation is indicated in the first PBD domain. In the second PBD domain, both H83D and H86D mutations are indicated by Xs. **B**) Normalized fluorescence excitation and emission spectra show a 1.53 fold difference between the constitutively active versus the dominant negative (not bound to GDI) versions of the Cdc42 biosensor. **C**) Normalized FRET/mCer ratios of wild-type (WT) and mutant forms of the Cdc42 biosensor with or without co-expression with negative regulator (GDI). **D**) and **E**) Normalized FRET/mCer ratios of wild-type biosensor co-expressed with upstream Cdc42 targeting and non-specific regulators (GEFs) (D) and negative regulators (GAPs) (E). Data in all cases are normalized to FRET/mCer ratio of wild-type biosensor alone. Data are the mean −/+ SEM of 3 different experiments. * p< 0.017, ** p<0.002, ***p<0.0006, ****p<0.0001, ns: non-significant. Significance designations on top of bars are compared to the wild-type biosensor expression alone. All other significance comparisons are specifically indicated.

Our laboratory has recently developed a fully genetically-encoded, single-chain, FRET-based Rac1 biosensor using monomeric Cerulean (mCer) and monomeric Venus (mVen) as the FRET pair (unpublished data). We have now extended this approach to create a new, genetically encoded, single-chain biosensor for Cdc42 (Fig.1A). The biosensor for Cdc42 incorporates mCer at the N-terminus, followed by two tandem p21 binding domains (PBD) derived from PAK1 with a structurally optimized linker in between the two PBDs and mVenus followed by full-length, wild-type Cdc42 at the C-terminus. Importantly, this design leaves the C-terminal hypervariable region and the prenylation motif of Cdc42 intact, thus accessible for correct membrane localization and regulation by GDI. The two PBDs have distinct functional roles in the biosensor: PBD1 modulates FRET response by interacting with the built-in Cdc42 in the GTP-loaded state, while PBD2 serves to auto-inhibit PBD1 to minimize FRET in the OFF state of the biosensor. PBD2 contains a set of GTPase-binding deficient mutations (H83D and H86D) to prevent interaction with the built-in or other endogenous GTPases, and to limit its function to the autoinhibition of PBD1. FRET level in the OFF state was further optimized by modulating the binding affinity of PBD1 to Cdc42 by including the H86D mutation in PBD1. Finally, the FRET dipole coupling angle between mCer and mVen has been optimized by incorporating a circular permutant of mVenus, monomeric cp229Venus [12].

As the size of the biosensor precluded *in vitro* purification, we tested and characterized the biosensor in HEK293 as previously described [13], [14]. Wild-type (wt) or mutant versions of the biosensor was overexpressed in HEK293 cells and the fluorescence emission spectra between 450 – 600 nm was measured in adherent cells upon excitation at 433 nm. To demonstrate proper response by the biosensor, mutations in Cdc42 were introduced that either activate (constitutively active G12V or Q61L) or inactivate (dominant negative T17N) the biosensor. The Cdc42 biosensor showed an approximate 75% increase in FRET ratio between inactive (T17N) versus the active (G12V) state, shown in Fig.1B. We tested the regulation of the biosensor by the negative regulator GDI as well. Since high levels of biosensor expression overwhelmed endogenous GDI [14], [15], addition of exogenous GDI was titrated to the lowest levels required for maximal inhibition of the biosensor (Fig.S1). This level of excess GDI produced nearly maximal reduction in FRET ratio of the G12V version of the biosensor to a similar level seen with the inactive biosensor, shown in Fig.1B. Excess GDI also reduced FRET levels of the wt biosensor, but not the Q61L mutant that is incapable of binding to GDI [16] (Fig.1C). Furthermore, a combination of effector-binding mutations in Cdc42, T35S and Y40C, or the additional GTPase-binding deficient H83D mutation in PBD1 (2× PBD) reduced FRET activity as expected (Fig.1C; Fig.S2A). The GDI-binding deficient mutant version (R66E) of the biosensor with or without excess GDI co-expression showed no difference in FRET/mCer ratio compared to the wt biosensor expression alone (Fig.S2B). The combination of the effector binding deficient (Y40C/T35S) and the GDI binding deficient (R66E) mutations reduced FRET activity to the same level with or without excess GDI co-expression (Fig.S2B). The difference between the GDI-bound (Fig. 1C: wt, G12V, or Y40C/T35S + excess GDI) versus the inactive but GDI-free (Fig. 1C: T17N) or that which is GDI-free but cannot bind effectors (Fig. 1C: Y40C/T35S) showed approximately 17% difference in FRET/mCer ratio. This significant and measurable difference is present using fluorometry where we overexpress mutants and regulators to drive specific interactions. However, it is also important to note that 90–95% of cellular Rho GTPases are found in complex with GDI in cytoplasm under normal conditions and thus only a small subset of GTPases would be free of GDI at any given time [17]–[19]. Also, in traditional microscopy imaging we measure ensemble averages of populations of biosensors undergoing different extents of FRET (on vs. off) at any given pixel, thus it would not be possible to directly determine the GDI-bound status of Cdc42 by simply inspecting the ratio values at different subcellular locations. The ability of fluorometry to distinguish these subtle differences further illustrates the ability of our biosensor to directly sense the full range of signal modulation, from the GDI-bound state to the fully activated state, ranging in the FRET/mCer ratio difference of up to 2.14 fold (Fig. 1C, comparing constitutively active versus the GDI bound; or Fig. 1D, comparing the wt + GDI versus the wt + Cdc42-targeting GEF). The inhibitory effects of excess GDI was rescued by co-expressing constitutively active versions of guanine nucleotide exchange factors (GEF) that act on Cdc42 (Dbs, Vav2 and ITSN2) resulting in increased FRET similar to that of wt biosensor overexpression without GDI (Fig.1D). However, GEFs that act on Rho (p190RhoGEF and Tim) or on Rac1 (Tiam1 and TrioGEF) failed to increase FRET levels as expected. GEFs that act on Cdc42 were also able to fully activate the biosensor in absence of the exogenous excess GDI co-expression, to levels similar to overexpression of the constitutively activated versions of the biosensor (Fig.1D). In addition, the biosensor exhibited proper responses in the presence of GTPase-activating proteins (GAP) including p50RhoGAP which reduced FRET levels similarly as excess GDI, while the non-Cdc42-targeting Rap1GAP had no effect (Fig.1E). One major concern is that exogenous biosensor expression may cause dominant-negative effects on the cell due to the possible competition with endogenous Cdc42 for downstream endogenous effectors. To demonstrate that the biosensor does not compete for endogenous effector binding, a GST-pulldown assay was performed using constitutively active Q61L biosensor containing either competent or non-binding PBD1 domain. The biosensor was detected in the pull-down fraction only when it contained the non-binding mutant PBD1 (Fig. S3), confirming that the active biosensor would not engage in spurious interactions with endogenous effector proteins.

**Figure 2:**
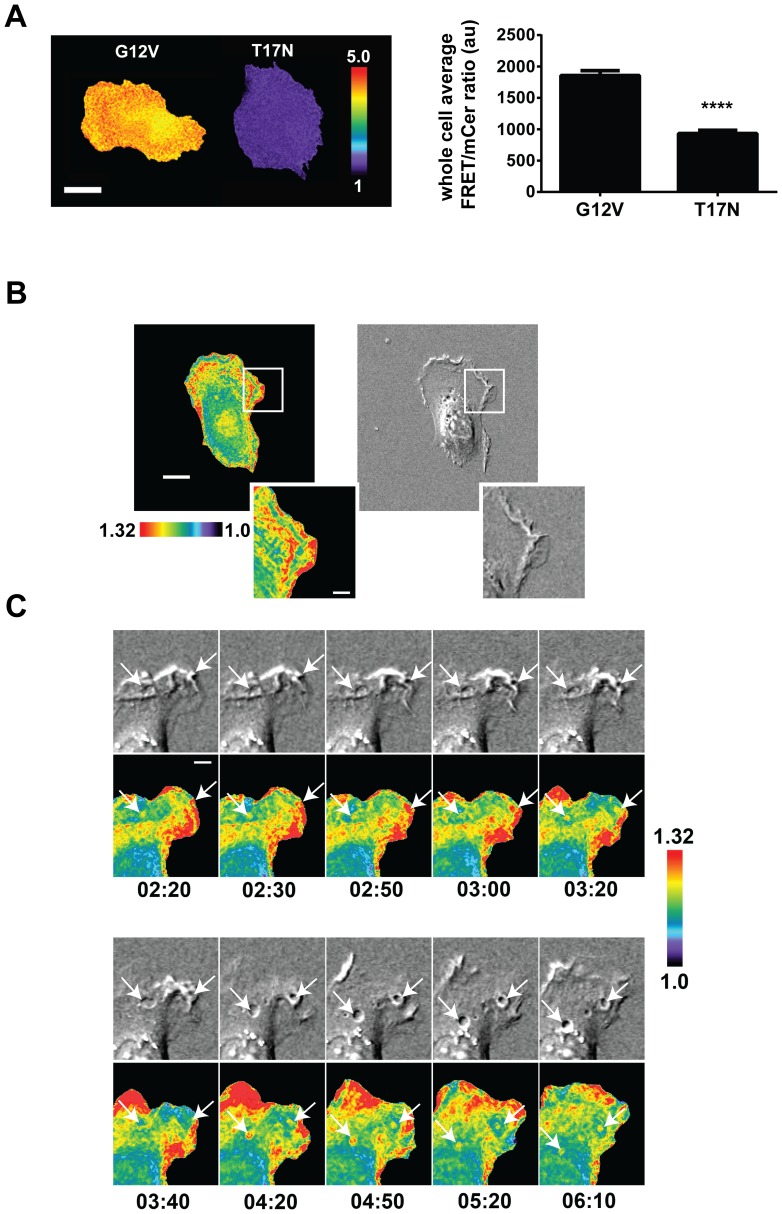
Cdc42 biosensor in mouse embryonic fibroblasts. **A**) A constitutively active (G12V) versus the dominant negative (T17N) versions of the Cdc42 biosensor was transiently overexpressed in MEFs, fixed, and imaged to calculate the whole-cell average ratio values. Scale bar = 20 µm. Quantification is also shown, n = 15 cells each, error limits are SEM, **** p<0.0001 using the student-t test. **B**) Representative still-frame of Cdc42 activation patterns at the edges of MEFs during random protrusion/retraction. The white bar in the whole-cell frame corresponds to 15µm, in the zoomed insets, 5µm. **C**) Zoomed panels of the timelapse of macropinocytosis are shown. White arrows point to the regions of interest, the bar indicates 5µm. Images shown are from widefield microscopy thus at the macropinosome, projections from multiple membrane layers are visible.

We next sought to further validate the new Cdc42 biosensor in mouse embryonic fibroblasts (MEF) using high-resolution imaging. The constitutively active (G12V) and the dominant negative (T17N) versions of the biosensor when transiently overexpressed in MEFs showed approximately 50% difference in the whole-cell average FRET/mCer ratio (Fig.2A), recapitulating the fluorometric measurements observed with the biosensor in HEK293 cell line (Fig.1). We then produced MEFs stably incorporating the Cdc42 biosensor under the tet-OFF inducible system as previously described [13], [20], and imaged the cells randomly protruding over fibronectin coated coverslips (Fig.2B; Movie S1, S2). Here, we observed robust and rapid turnover of edge protrusion/retraction and associated Cdc42 activation patterns. Furthermore, we observed activation dynamics of Cdc42 during macropinocytosis occurring at the edge of cells during protrusion and membrane turnover (Fig.2C; Movie S3). Cdc42 activity appeared to be dynamically modulated (Fig.2C) and remained elevated once the macropinosome was engulfed and as it travelled through the cell body. The patterns observed here appears to be different than the associated dynamics previously observed using biosensors for other Rho family proteins [14], [21] and appears to suggest the involvement of Cdc42 in macropinocytosis.

**Figure 3:**
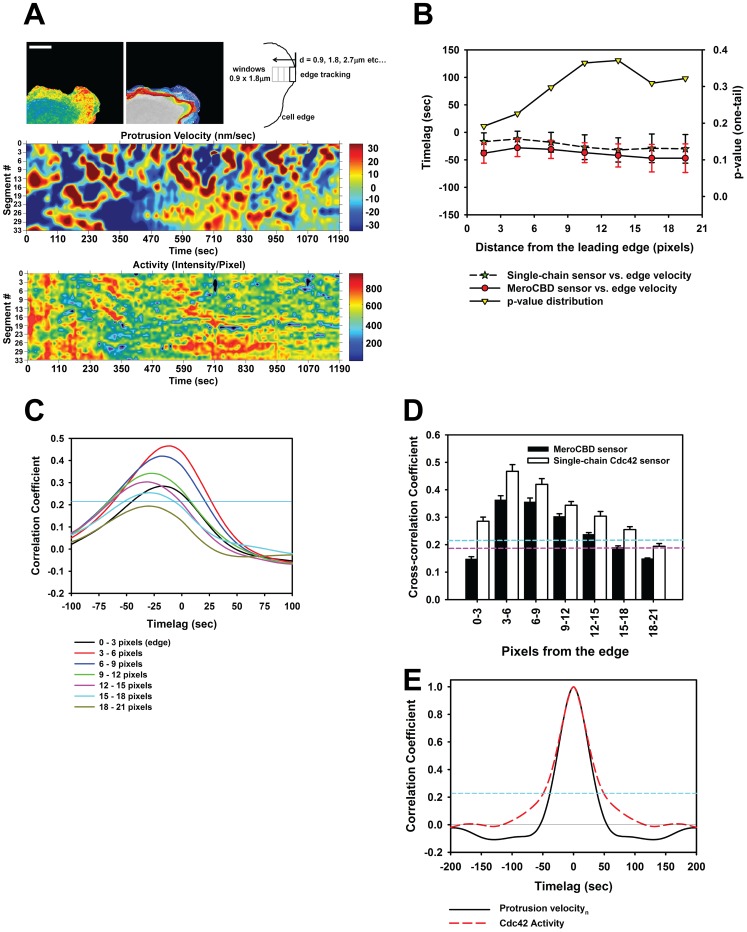
Morphodynamics analysis comparing the new Cdc42 biosensor to MeroCBD. **A**) A representative edge segment is shown together with the temporal evolution of the edge location and the sampling window construction scheme. Below, the morphodynamics mapping of the edge velocities and the associated Cdc42 activation fluctuations are shown. **B**) The comparison of the cross-correlational timelags at different spatial locations are shown for the new Cdc42 biosensor and the MeroCBD, shown with SEM. P-values are also shown. MeroCBD: n = 8 cells: 414 windows; single-chain Cdc42 biosensor: n = 9 cells: 532 windows. **C**) The evolution of the timelags as a function of the spatial position away from the edge for the new single-chain biosensor. Region above the blue line indicates p<0.05. **D**) Comparison of the strength of the cross-correlation coupling between the edge velocity and the associated Cdc42 at different spatial location, shown for both MeroCBD and the new single-chain biosensor. The region above the dashed blue line indicates the p<0.05 for the single-chain sensor and the dashed purple line indicates that for the MeroCBD sensor. **E**) Autocorrelation functions for the normal-direction edge velocity and Cdc42 activity measured using the new single-chain biosensor. The region above the blue dashed line indicates p<0.05.

Next we sought to determine if the new single-chain Cdc42 biosensor would recapitulate the activation dynamics and the kinetic/kinematic coupling during random fluctuations of the leading edge, measured previously using the MeroCBD biosensor in MEFs [6], [11]. Here, we applied the same computational methods for the analysis of the leading edge dynamics as previously described [11]. We measured the dynamics of Cdc42 activation and edge protrusion velocities within sampling windows of 0.9 × 1.8 µm (3 × 6 pixels) constructed along the leading edge (Fig.3A) and tracked the edge movement during random protrusive events as previously performed. The cross-correlational timelags between our new single-chain Cdc42 and the previously published MeroCBD were statistically indistinguishable within the distances of 0 – 6.3µm from the leading edge (Fig.3B). The strength of the coupling between the Cdc42 activation dynamics and the kinematics of the edge reached a maximum at 0.9 – 1.8µm from the leading edge, and decreased from this spatial position in both proximal and distal directions as previously reported (Fig.3C) [11]. The timelags between the protrusion and the measured Cdc42 activity also shifted into increasingly negative delay with decreasing cross-correlation coefficient values, suggested a mechanism of diffusive transport of activated Cdc42 from the region just behind the edge (strongest coupling at 0.9–1.8µm) to regions away from the edge, as previously observed (Fig.3D) [11]. Additionally, the autocorrelation functions of edge motion and Cdc42 activation cycling at the edge appeared to be virtually identical to the previously published MeroCBD results [11] (Fig.3E). Overall, this data indicates that our new Cdc42 single-chain biosensor reports activation dynamics similar to measurements of endogenous Cdc42 activity using the MeroCBD biosensor system [6], [11].

**Figure 4:**
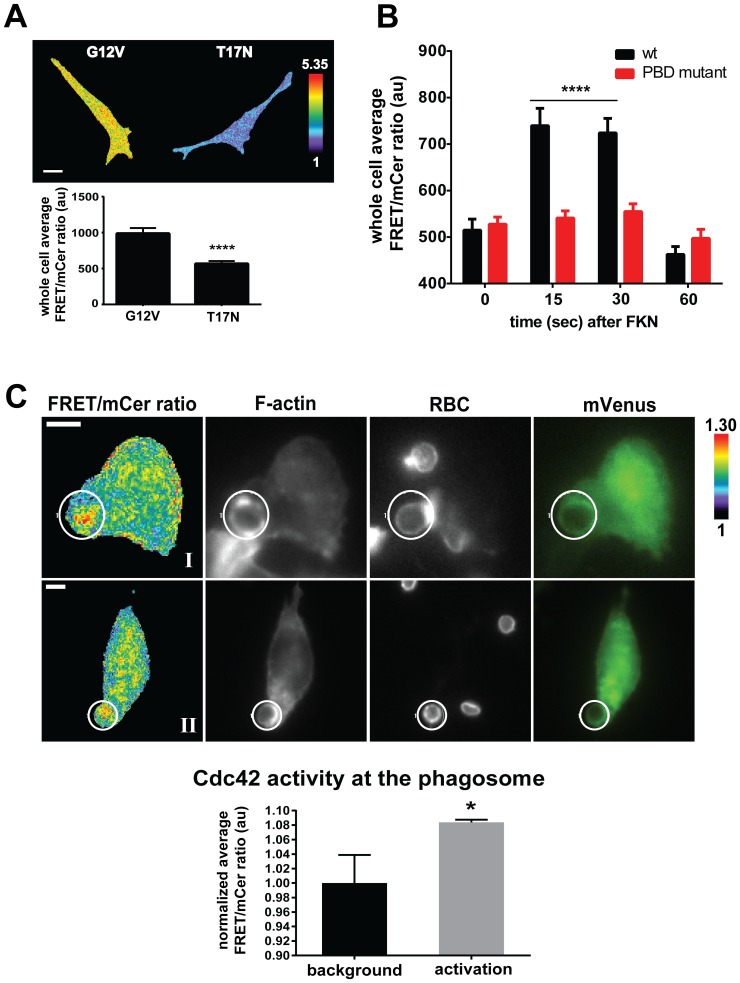
Cdc42 is activated during macrophage phagocytosis and CX3CL1 stimulation. **A**) Whole cell average of FRET/mCer ratio analyzed in RAW/LR5 cells transiently expressing G12V or T17N mutant forms of the Cdc42 biosensor. Scale bar = 10 µm. n = 15 cells per condition with mean +/− SEM, **** p<0.0001. **B**) Quantitation of whole cell average of FRET/mCer ratio analyzed in RAW/LR5 cells transiently expressing wild-type or PBD mutant control of Cdc42 biosensor and stimulated with 50 ng/mL CX3CL1 for indicated times. Average raw values of 3 independent experiments and at least 15 cells per condition per experiment with SEM, **** p<0.0001 compared to wt at t = 0 time point. No significant changes were measured for the PBD mutant set. **C**) Representative ratiometric images of RAW/LR5 cells transiently expressing wild-type Cdc42 biosensor (Ratio), F-actin, Red blood cells (RBC) (grayscale panels), mVenus-biosensor localization (green panels). The white bar in the whole-cell frame corresponds to 10 µm. Quantitation of active levels of Cdc42 at the phagosome (designated by white circle) normalized to background levels are shown; n = 18 phagocytic events, values are mean +/− SEM, * p<0.0193. Further localization analysis of these representative cells are shown in Fig.S4.

We next sought to demonstrate the usefulness of our new biosensor in reporting Cdc42 activity associated with biological functions known to require Cdc42 activity and we chose macrophages as a model system. Cdc42 is required for critical macrophage functions including phagocytosis and chemotaxis [22]–[24]. First, we transiently overexpressed constitutively active (G12V) or dominant negative (T17N) mutants of the biosensor in the RAW/LR5 monocytic/macrophage cell line [25]. The biosensor showed approximately 40% difference in the whole-cell average of FRET/mCer ratio between active and inactive states (Fig.4A), consistent with our measurements in MEFs and in fluorometry. We then performed a set of experiments to functionally validate the wild-type Cdc42 biosensor in macrophages based on previously published observations of important macrophage functions. We stimulated cells transiently overexpressing the wild-type biosensor with CX3CL1 (Fractalkine), a potent macrophage chemoattractant [26], to determine if measurements with ratiometric imaging of Cdc42 biosensor would be consistent with the activation dynamics of Cdc42 previously shown by traditional GST-pulldown assay [24]. In the cells expressing the wild-type biosensor we detected maximal Cdc42 activation by 15 sec of stimulation that leveled off by 1 min, shown in Fig.4B, similarly as determined by Park H *et al* [24]. The biosensor response to CX3CL1 was specific as we did not measure any significant activation over time using the inert PBD mutant biosensor. Next we tested the response of the Cdc42 biosensor towards IgG-opsonized particles. Hoppe *et al.* had previously demonstrated localized Cdc42 activity during macrophage phagocytosis using a dual-chain, intermolecular biosensor system during live-cell imaging and reported that Cdc42 activity appears by 1 min, during phagocytic cup formation and is sustained until phagosome closure, estimated at 7–9 min [27]. Based on these published observations we proceeded to examine Cdc42 activity at the early phase of phagosome formation. We transiently overexpressed the wild-type biosensor and performed synchronized phagocytosis. Figure 4C shows representative ratiometric images of localized Cdc42 activity at F-actin-rich phagosomes at the F-actin focal plane. We measured an average increase in Cdc42 activity of 8.37% at the phagosome with 81.8% (18/22) of the phagocytic events demonstrating Cdc42 activity. To further analyze the relative localization of Cdc42 activity to F-actin, we created binary masks (see methods) for each component of the phagosome (FRET/mCer ratio, F-actin and RBC) which were overlayed in pairs (shown in Fig.S4). This analysis showed that Cdc42 activity was present at a defined interface with F-actin, with some or little co-localization, in agreement with the results from Hoppe *et al* [27]. Because phagocytic cups form three-dimensional structures we wished to determine the Cdc42 activity throughout the phagosome by measuring the activity at different Z-positions. Fig. S5 shows an example of ratiometric measurements at different Z-stack positions, illustrating differential patterns of Cdc42 activation within a phagosome. Overall, these results demonstrated that our new single-chain Cdc42 biosensor yields ratiometric measurements consistent with previously published results using different methods. Additionally, there appeared to be no adverse effect on the number of phagocytic events induced by overexpression of the biosensor with an average of 1.6 phagocytic cups/cells observed in both expressing and non-expressing cells (p = 0.900, n≥22). These observations further suggest that expression of the biosensor appeared to have no functional dominant negative effect.

**Figure 5:**
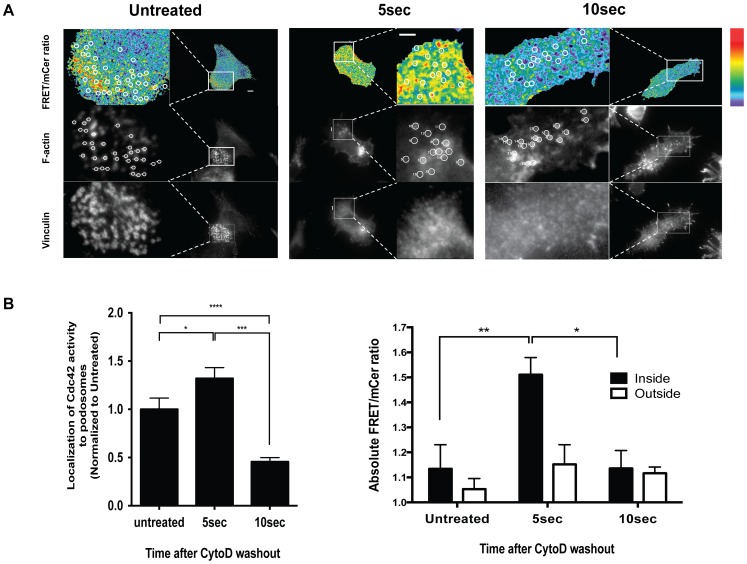
Localized Cdc42 activity during podosome reformation. **A**) Representative images of cells expressing Cdc42 biosensor left untreated, or treated with Cytochalsin D for 10 min and then washed for 5 sec and 10 sec before fixation and staining for F-actin and vinculin. Inset shows the zoomed view of the podosome structures. Regions shown with white circles in the F-actin stain, were used to determine the associated Cdc42 activity levels for quantitation inside podosomes. Regions next to the drawn circles were used to quantitate activity outside podosomes. The white bar in the whole-cell frame corresponds to 10µm, in the zoomed insets, 5µm. The linear pseudocolor lookup table (black to red) for the FRET/mCer ratio ranges correspond to a range of 1.0 – 1.77. **B**) Left: Quantitation of the number of co-localized Cdc42 activation spots were scored from (A) normalized to untreated cells. Right: Quantitation of Cdc42 activity in regions inside (black bars) versus outside podosomes (clear bars). n≥8 cells from 3 independent experiments, data represent mean −/+ SEM. *p<0.0101, **p<0.0064, ****p<0.0001.

Additionally, we examined Cdc42 activity at actin-rich podosome structures. Podosomes are crucial for many macrophage functions including cellular adhesion and matrix degradation, have high turnover rates that allow quick adaptation to extracellular environment[28]. Previously, it was reported that activation of WASp by Cdc42 was required to regulate the formation of podosomes in primary macrophages [29], however, expression of a constitutively activated version of Cdc42 led to podosome disassembly [29]. Moreover, it is currently unknown if Cdc42 activity is actually localized to podosomes. To examine Cdc42 activity at podosomes, RAW/LR5 cells were transiently transfected with the Cdc42 biosensor. Figure 5A shows ratiometric images for localized Cdc42 activity at F-actin-rich podosome structures. Surprisingly under steady-state conditions, only a subset of the podosomes demonstrated localized Cdc42 activity. Podosomes are dynamic structures that are constantly being remodeled and this result suggested that Cdc42 may not be required during the entire lifetime of a podosome. To examine podosome initiation cells were treated with Cytochalasin D for 10 min to dissolve the preexisting podosomes, and then the Cytochalasin D was removed to allow for the synchronous reformation of podosomes. As shown in Figure 5A, we observed a burst of high Cdc42 activity localized to podosome structures at 5 seconds following the Cytochalasin D removal. This activity was correlated to an increase in regions inside actin-rich podosomes as compared to regions immediately outside. However, this activity diminished significantly at 10 seconds after the Cytochalasin D washout (Fig. 5B). These results show for the first time, a direct observation of transient Cdc42 activity during podosome reformation. Many studies have shown the importance of Cdc42 in podosome formation [23], [29]. In fact, Cdc42 is required for the efficient phosphorylation of WASp by tyrosine kinases. This phosphorylation is important for proper targeting of active WASp to podosomes [22], [30], [31]. Using our new biosensor we were able to detect a high Cdc42 activity at podosomes initially as they are being assembled which then drops in the time scale of seconds. This suggests an intriguing possibility that Cdc42 could be required for the initial activation of WASp and might not be necessary for sustaining this activity. Whether Cdc42 undergoes a cycle of activation and inactivation states during podosomes turnover dynamics requires further investigation. However, this observation highlights the importance of our new Cdc42 biosensor in sensing a localized, transient activation pattern of Cdc42 during the formation of these dynamic cellular structures.

Here, we described our new single-chain, genetically encoded biosensor for Cdc42, based on FRET. Our biosensor incorporates a full-length Cdc42 at the C-terminus of the molecule to allow for post-translational lipid modification, critical for correct interactions with appropriate membrane compartments and with the upstream regulator GDI. Previous approaches, in which the Cdc42 was designed into either the N-terminus of the molecule [8] or the internal portion of the biosensor [9], artificially induced plasma membrane localization through attachment of k-ras CAAX-box. These designs produced GEF/GAP sensors that do not fully reflect the regulatory cycle of the GTPase including interaction with the GDI, important for the shuttling between the membrane and the cytoplasmic compartments. Furthermore, bi-molecular approaches in which the FRET donor/acceptor halves are present in two separate molecules [11], [32] are often difficult to correctly interpret due to non-equimolar distribution of the components throughout cells. The intra-molecular design that we incorporate here, assures an equimolar distribution of the FRET donor/acceptor components making the data interpretation more straightforward. Our new biosensor compares favorably to meroCBD, a previous generation sensor for detection of endogenous Cdc42 activity based on solvatochromic dyes [6], but side-steps the often cumbersome *in vitro* preparation and microinjection accompanying this previous approach. We have set forth here to validate our new biosensor against previously reported results from MEFs as well as in macrophage cell line, and show that indeed, we observe appropriate activation patterns and changes in activation levels during leading edge protrusion and retraction, and during phagocytosis and CX3CL1 stimulation [11], [27], [33]. Importantly, the exogenous stimulation using CX3CL1 showed an identical time course of Cdc42 activation to that previously reported using the traditional affinity pull-down method[33], as well as using the meroCBD biosensor to measure the activation changes in the endogenous Cdc42 following CX3CL1 stimulation in RAW/LR5 cells [34]. These results show that the new single-chain biosensor for Cdc42 we report here faithfully reproduces the endogenous Cdc42 activation kinetics measured using different, proven techniques and thus is a useful tool for detecting the activity of Cdc42 in living cells.

## Materials and Methods

### Cdc42 biosensor and fluorometry

Cdc42 biosensor was created by linking monomeric Cerulean [35] to two tandem p21 binding domains (PBD) of p21 activated kinase 1, amino acid residues 70–149, separated by a structurally optimized linker (GSGGPPGSGGSG), a circularly permutated monomeric Venus [12], and a full-length wildtype Cdc42 (Fig.S6). The second PBD (PBD2) contained two point mutations to render it unable to bind to active GTPase (H86/83D mutation) [36]. In addition, the first PBD (PBD1) contained a single point mutation (H86D) to fine tune the affinity towards activated Cdc42 to achieve reduced FRET/mCer ratio for the dominant negative mutant version of the biosensor while maintaining robust and high FRET/mCer ratio for the constitutively active mutant version of the biosensor or the overexpression of the wildtype Cdc42 version of the biosensor. The biosensor cDNA cassette was subcloned into pTriEX-HisMyc4 (Novagen) for transient expression. Characterization of biosensor response was performed in HEK293 cells overexpressing the biosensor with or without the appropriate upstream regulators, and a plate-reader was used to measure the fluorescence response in the spectrofluorometer (Horiba-Jobin-Yvon Fluorolog-3MF2 with MicroMax plate reader), as described previously[13], [14]. Briefly, HEK293 cells were overnight plated at 1.25×10^6^ cells/well of 6-well plates coated with poly-L-lysine, and transfected using Lipofectamine2000 (Invitrogen) following the manufacturer's protocols. The biosensor and the regulator cDNAs were co-transfected at ratios of 1∶3 for the biosensor and the GDI or the GAP and 1∶3∶1 – 10 for the biosensor:GDI:GEF. Adherent cells were washed in PBS and fixed using 3.7% formaldehyde forty eight hours following the transfection, and then the well plates containing fixed cells were placed into the plate reader to measure fluorescence emission spectra. The spectra were obtained by exciting with 433 nm light, with emission scanned from 450 – 600 nm. The fluorescence reading of a sample cell suspension with empty cDNA (pCDNA3.1) was used to measure light scatter and autofluorescence, which were subtracted from the data. The resulting spectra were normalized to the peak mCer emission intensity at 475 nm to generate the final ratiometric spectra.

### Cell culture

MEF/3T3 tet-OFF (Clontech) were cultured in Dulbecco's modified Eagle's medium (Mediatech) with 10% FBS. A stable cell line expressing inducible Cdc42 biosensor was produced using the pRetro-X-Puro tet-inducible retroviral system as previously described [13], [20]. To repress the biosensor expression during normal culture, 2µg/ml doxycycline was applied. To induce Cdc42 biosensor expression, doxycycline was removed 48 hours prior to imaging by detaching cells through brief trypsinization and then replating at 1×10^4^ cells per 10 cm dish. Cells were plated on fibronectin (10 µg/ml)-coated glass coverslips for 2 hours prior to imaging. Imaging was performed in Ham's F-12K without phenol red (Crystalgen) with 3% FBS in a heated closed chamber. RAW/LR5 cells are derivative of monocyte/macrophage RAW 264.7 cells [25], cultured in RPMI 1640 supplemented with 10% newborn calf serum, 100 U/mL penicillin and 100 µg/mL streptomycin, maintained at 37°C and 5% CO_2_ atmosphere. Biosensor constructs were transiently expressed in RAW/LR5 cells via transfection with FugeneHD (Fisher Scientific) according to the manufacturer's instructions: cells were plated in a 12-well plate the day before transfection; next day cells (70–90% confluent) were incubated in the transfection mix for 2 hrs, then replated onto 12-mm glass coverslips (in a 24-well plate) and incubated overnight at 37°C and 5% CO_2_ atmosphere.

### Exogenous stimulation and phagocytosis

RAW/LR5 cells were transiently transfected with the wild-type or PBD mutant Cdc42 biosensor as above. For exogenous stimulation, cells were serum-starved in RPMI for at least 4 hrs prior to stimulation with 50 ng/mL CX3CL1 in BWD (125 mM NaCl, 5 mM KCl, 1 mM KH_2_PO_4_, 5 mM glucose, 10 mM NaHCO_3_, 1 mM MgCl_2_, 1 mM CaCl_2_, and 20 mM Hepes) for indicated times at 37°C before fixation. For synchronized phagocytosis, cells were cooled on ice for ∼ 5 min. Then media was replaced with ice-cold BWD, ∼2.5–5 × 10^6^ rabbit IgG-opsonized sheep red blood cells (RBC) were added and let sit on ice for 15 mins to allow binding of RBCs to cells. Cells were rinsed three times with ice-cold BWD to wash away unbound RBCs and coverslips were transferred to BWD pre-warmed to 37°C (in water bath) and incubated for 1 min before fixation. Cells were fixed in 3.7% formaldehyde in BWD, permeabilized, and stained for RBCs with Alexa Flour 568 anti-rabbit IgG antibody and F-actin with Alexa Flour 568-phalloidin (CX3CL1 stimulation) or 680-phalloidin (phagocytosis). For ratiometric imaging cells were mounted in 50% glycerol in PBS. To analyze Cdc42 activity and localization during phagocytosis, top 15% of the FRET/mCer ratio image was thresholded to create a binary mask. The FRET/mCer image was multiplied by its binary mask to isolate the top 15% of ratio intensity. The resultant image was then subtracted from FRET/mCer ratio image to isolate the bottom 85% of ratio intensity. In these “top” and “bottom” ratio images a region corresponding with F-actin and RBC was selected (designated by circle shown in Fig. 4C) to measure the average FRET/mCer ratio levels in the top and bottom ranges. The average values for n = 18 were normalized to show background and activation levels. Binary masks were also created for the corresponding images of F-actin and RBC staining by thresholding intensity levels that selected the majority of fluorescent intensity positive regions. Binary masks of top 15% FRET/mCer ratio and its corresponding F-actin staining were overlayed to analyze relative localization of Cdc42 activity to F-actin at the phagosome.

### Podosome reformation assay

RAW/LR5 cells were transfected with the wildtype Cdc42 biosensor for 2 hours and then plated overnight on fibronectin-coated coverslips. Cells were serum-starved for 4 hours prior to the assay. For podosomes reformation and synchronization, cells were incubated with Cytochalasin D (Sigma) at a final concentration of 2µM for 10 minutes, resulting in complete dissolution of podosomes. Cytochalasin-D-containing medium was then removed and replaced with warm serum-free RPMI supplemented with 25 mM HEPES, pH 7.4 for the indicated times. Cells were fixed, permeabilized and stained for F-actin with Alexa Fluor 680-phalloidin (Invitrogen) and vinculin (anti-h-VIN1) (Sigma) followed by secondary antibody containing Alexa Fluor-568 (Invitrogen). For ratiometric imaging, cells were mounted in 50% glycerol in PBS. For quantitation, ratio was taken between the average highest and lowest intensities of all images in all three cases. Regions of F-actin corresponding to podosome structures were used to measure the associated Cdc42 intensity ‘inside podosomes’ in the ratiometric images. Regions immediately adjacent were used to measure the associated Cdc42 intensity ‘outside podosomes’. The average ratio value between highest and lowest intensities within the average scale range was set as a conservative threshold ratio value above which Cdc42 activity was scored as ‘active’. The number of active spots localized to podosome structures was quantified in all three conditions (untreated, 5 sec and 10 sec) and then normalized to the untreated condition.

### Microscopy imaging

Activations of Cdc42 biosensor were measured by observing the ratio of FRET emission to mCerulean emission. For live cell experiments using MEF/3T3 cells, images were acquired at 40× magnification (Olympus UIS2 40× N/A 1.3) using a custom microscope capable of simultaneous acquisitions of FRET with mCerulean emission channels, through two CoolsnapES2 cameras mounted via a beamsplitter [37]. Images simultaneously acquired were properly aligned for a pixel-by-pixel matching using *a priori* calibration and morphing to achieve accurate registration prior to ratiometric calculations as described previously [38]. Image processing including flat-field correction, background subtraction, ratio calculations and correction for photobleaching were performed as described previously [38]. For fixed cell imaging of macrophages, a single CoolsnapHQ2 camera attached on the bottom 100% throughput port of the microscope was used together with a 60× magnification objective lens (Olympus UIS2 60× N/A 1.45). In this case, excitation and emission filterwheels allowed for the switching of appropriate filter sets to acquire mCerulean, mVenus, and FRET emissions. The optical specifications for this configuration of the system used are detailed elsewhere [39]. In both live-cell imaging and fixed-cell imaging, transilluminated images were also obtained. For imaging of fixed macrophages overexpressing the biosensor, we controlled for the relative expression levels by imaging only those cells that had brightness to fill approximately 80% of the 12-bit digitization range of the CCD chip, using excitation intensities of 0.4–1.0 mW at the specimen plane.

### Edge tracking and correlation analysis

The protrusion tracking algorithm was used to track and construct the measurement windows as previously described [11]. Briefly, the leading edge tracking was performed on a segment of a cell image stack that showed robust protrusion/retraction cycling. The cropped image stack was processed to track the edge motion using the prPanel.m protrusion tracking software [11]. The measurement windows of the size 3 by 6 pixels which translated to 0.927µm by 1.854µm at 40× magnification was shown previously to be the diffusion-limited area size for the 10 s time intervals of each successive time points of acquisition. Typically, the entire leading edge segment measured contained 30 – 100 measurement windows depending on the overall length of the segment. The windows' positions were successively moved back away from the leading edge in 3-pixel units, to calculate the spatial dependence of the cross-correlation functions. This was used to measure the correlational coupling up to 6.489µm distance from the leading edge. The normalized cross-correlation coefficient was computed at each window between the measured velocity in the normal direction at the edge and the changes in Cdc42 activity at the corresponding window using the matlab function *xcov*. The individual cross correlation coefficient distribution at each window was treated as an independent measurement entity, smooth-spline fitted, pooled between all cells imaged and the average maximal cross-correlation coefficient timelag location and the 95% confidence interval were calculated by a non-parametric bootstrap method [40].

## Supporting Information

Figure S1 **GDI titration effect on wild-type Cdc42 biosensor.** Normalized FRET/mCer ratios of wild-type biosensor (WT) with and without co-expression with increasing concentrations of negative regulator (GDI) starting from 1∶1 ratio up to 4 folds excess GDI.
(TIF)

Figure S2 **Characterization of GTPase-binding mutant of the Cdc42 biosensor. A)** Normalized FRET/mCer ratios of wild-type (WT) and mutant forms of the biosensor containing a second GTPase-binding deficient mutation in PBD domain (2xPBD) with or without co-expression with negative regulator (GDI). **p<0.0062, ***p<0.0007, ns: non-significant. **B)** Normalized FRET/mCer ratios of wild-type (WT) and mutant forms of the biosensor containing a GDI-binding deficient mutation R66E with or without co-expression with GDI, as well as a combination of effector binding and GDI-binding mutations R66E/T35S/Y40C with or without co-expression with GDI. *<0.0112, ns: non-significant.
(TIF)

Figure S3 **Cdc42 biosensor does not bind to endogenous effectors.** Western blot of GST-PAK pulldown of active forms of mCherry tagged-Q61L constitutively active Cdc42 mutant, T17N dominant negative and Q61L constitutively active version of the biosensor with competent or non-binding mutations in the PBD1 domain (left). Total lysates and β-actin was used as a loading control (right). Here, we show that the biosensor with competent PBD cannot be pulled down using excess exogenous effector, showing that the built-in binding domain cannot be competed away by the downstream effectors thus minimizing the dominant negative effect. The biosensor can be pulled down only if both PBD domains contain the GTPase binding mutations (H83D/H86D).
(TIF)

Figure S4 **Relative localization of Cdc42 activity to F-actin at the phagosome.** Zoomed view of phagosomes from image sets in Figure 4 are shown. Top and bottom panel sets correspond to cells I and II, indicated in the FRET/mCer ratio images. First column shows original images of FRET/mCer ratio, F-actin and RBC. Second column shows binary masks created for original images. In the third column, binary mask of F-actin was overlayed with that of FRET/mCer ratio to show the relative localization of Cdc42 activity to F-actin. Binary mask overlays of F-actin with RBC and RBC with FRET/mCer ratio are also shown. White scale bar = 1 µm.
(TIF)

Figure S5 **Ratiometric imaging of Cdc42 activity over serial planes of the phagosome.** RAW/LR5 cells transiently expressing wt Cdc42 biosensor were imaged **A)** at optimal F-actin focal plane as in Figure 4C and **B** **)** in Z-series at 1 µm-steps where the focal plane from A was set as the center (*). Planes 4-1 progress down towards the base of the phagocytic cup and below, while planes 6-8 move upwards from the F-actin plane. Shown is a representative image set of n = 6 cells. Scale bar for whole cell = 10 µm and zoomed inset = 1 µm. Imaged by oil/60X.
(TIF)

Figure S6 **Amino acid sequence and the domain structure of the new biosensor for Cdc42.** H86D mutation in the PBD1 is shown in blue, and H86/83D mutations in the PBD2 are shown in red.
(TIF)

Movie S1 **Cdc42 activity at the edge of a MEF cell plated on 10 µg/ml fibronectin.** Duration of original sequence: 20 min. Magnification 40×, 2×2 binning. Frame interval: 10 sec. Replay: 5 frames/sec. Scale bar: 10 µm. Color bar defines the dynamic range of the FRET/mCer ratio. See Figure 2B.
(MOV)

Movie S2 **Additional example of Cdc42 activity at the edge of a MEF cell plated on 10 µg/ml fibronectin.** Duration of original sequence: 10 min. Magnification 40×, 2×2 binning. Frame interval: 10 sec. Replay: 5 frames/sec. Scale bar: 10 µm. Color bar defines the dynamic range of the FRET/mCer ratio.
(MOV)

Movie S3 **Cdc42 activity and corresponding DIC of a MEF undergoing macropinocytosis.** Note burst of activation accompanying vesicle closure. Duration of original sequence: 16 min. Magnification 40×, 2×2 binning. Frame interval: 10 sec. Replay: 5 frames/sec. Scale bar: 5 µm. Color bar defines the dynamic range of the FRET/mCer ratio. See Figure 2C.
(MOV)

## References

[1] Johnson DI (1999) Cdc42: An essential Rho-type GTPase controlling eukaryotic cell polarity. Microbiol Mol Biol Rev 63: 54–105. 10066831 10.1128/mmbr.63.1.54-105.1999 PMC98957

[2] Tang Y, Olufemi L, Wang MT, Nie D (2008) Role of Rho GTPases in breast cancer. Front Biosci 13: 759–776. 17981586 10.2741/2718

[3] Cerione RA (2004) Cdc42: new roads to travel. Trends Cell Biol 14: 127–132. 15003621 10.1016/j.tcb.2004.01.008

[4] Stengel K, Zheng Y (2011) Cdc42 in oncogenic transformation, invasion, and tumorigenesis. Cell Signal 23: 1415–1423. 21515363 10.1016/j.cellsig.2011.04.001 PMC3115433

[5] Wennerberg K, Der CJ (2004) Rho-family GTPases: it's not only Rac and Rho (and I like it). J Cell Sci 117: 1301–1312. 15020670 10.1242/jcs.01118

[6] Nalbant P, Hodgson L, Kraynov V, Toutchkine A, Hahn KM (2004) Activation of endogenous Cdc42 visualized in living cells. Science 305: 1615–1619. 15361624 10.1126/science.1100367

[7] Hodgson L, Nalbant P, Shen F, Hahn K (2006) Imaging and photobleach correction of Mero-CBD, sensor of endogenous Cdc42 activation. Methods Enzymol 406: 140–156. 16472656 10.1016/S0076-6879(06)06012-5

[8] Seth A, Otomo T, Yin HL, Rosen MK (2003) Rational design of genetically encoded fluorescence resonance energy transfer-based sensors of cellular Cdc42 signaling. Biochemistry 42: 3997–4008. 12680752 10.1021/bi026881z

[9] Itoh RE, Kurokawa K, Ohba Y, Yoshizaki H, Mochizuki N, et al (2002) Activation of rac and cdc42 video imaged by fluorescent resonance energy transfer-based single-molecule probes in the membrane of living cells. Mol Cell Biol 22: 6582–6591. 12192056 10.1128/MCB.22.18.6582-6591.2002 PMC135619

[10] Roberts PJ, Mitin N, Keller PJ, Chenette EJ, Madigan JP, et al (2008) Rho Family GTPase modification and dependence on CAAX motif-signaled posttranslational modification. J Biol Chem 283: 25150–25163. 18614539 10.1074/jbc.M800882200 PMC2533093

[11] Machacek M, Hodgson L, Welch C, Elliott H, Pertz O, et al (2009) Coordination of Rho GTPase activities during cell protrusion. Nature 461: 99–103. 19693013 10.1038/nature08242 PMC2885353

[12] Nagai T, Yamada S, Tominaga T, Ichikawa M, Miyawaki A (2004) Expanded dynamic range of fluorescent indicators for Ca(2+) by circularly permuted yellow fluorescent proteins. Proc Natl Acad Sci U S A 101: 10554–10559. 15247428 10.1073/pnas.0400417101 PMC490022

[13] Hodgson L, Pertz O, Hahn KM (2008) Design and optimization of genetically encoded fluorescent biosensors: GTPase biosensors. Methods Cell Biol 85: 63–81. 18155459 10.1016/S0091-679X(08)85004-2

[14] Pertz O, Hodgson L, Klemke RL, Hahn KM (2006) Spatiotemporal dynamics of RhoA activity in migrating cells. Nature 440: 1069–1072. 16547516 10.1038/nature04665

[15] Fritz RD, Letzelter M, Reimann A, Martin K, Fusco L, et al (2013) A versatile toolkit to produce sensitive FRET biosensors to visualize signaling in time and space. Sci Signal 6: rs12. 23882122 10.1126/scisignal.2004135

[16] Michaelson D, Silletti J, Murphy G, D'Eustachio P, Rush M, et al (2001) Differential localization of Rho GTPases in live cells: regulation by hypervariable regions and RhoGDI binding. J Cell Biol 152: 111–126. 11149925 10.1083/jcb.152.1.111 PMC2193662

[17] Garcia-Mata R, Boulter E, Burridge K (2011) The ‘invisible hand’: regulation of RHO GTPases by RHOGDIs. Nat Rev Mol Cell Biol 12: 493–504. 21779026 10.1038/nrm3153 PMC3260518

[18] Boulter E, Garcia-Mata R, Guilluy C, Dubash A, Rossi G, et al (2010) Regulation of Rho GTPase crosstalk, degradation and activity by RhoGDI1. Nat Cell Biol 12: 477–483. 20400958 10.1038/ncb2049 PMC2866742

[19] Ren XD, Kiosses WB, Schwartz MA (1999) Regulation of the small GTP-binding protein Rho by cell adhesion and the cytoskeleton. EMBO Journal 18: 578–585. 9927417 10.1093/emboj/18.3.578 PMC1171150

[20] Hodgson L, Shen F, Hahn K (2010) Biosensors for characterizing the dynamics of rho family GTPases in living cells. Curr Protoc Cell Biol Chapter 14: Unit 14 11 11–26. 10.1002/0471143030.cb1411s46 PMC2998069 20235099

[21] Zawistowski J, Sabouri-Ghomi M, Danuser G, Hahn K, Hodgson L (2013) A RhoC biosensor reveals differences in the activation kinetics of RhoA and RhoC in migrating cells. Plos One In Press. 10.1371/journal.pone.0079877 PMC3818223 24224016

[22] Park H, Cox D (2009) Cdc42 regulates Fc gamma receptor-mediated phagocytosis through the activation and phosphorylation of Wiskott-Aldrich syndrome protein (WASP) and neural-WASP. Mol Biol Cell 20: 4500–4508. 19741094 10.1091/mbc.E09-03-0230 PMC2770938

[23] Dovas A, Gevrey JC, Grossi A, Park H, Abou-Kheir W, et al (2009) Regulation of podosome dynamics by WASp phosphorylation: implication in matrix degradation and chemotaxis in macrophages. J Cell Sci 122: 3873–3882. 19808890 10.1242/jcs.051755 PMC2773189

[24] Park H, Cox D (2011) Syk regulates multiple signaling pathways leading to CX3CL1 chemotaxis in macrophages. J Biol Chem. 10.1074/jbc.M110.185181 PMC3083178 21388954

[25] Cox D, Chang P, Zhang Q, Reddy PG, Bokoch GM, et al (1997) Requirements for both Rac1 and Cdc42 in membrane ruffling and phagocytosis in leukocytes. J Exp Med 186: 1487–1494. 9348306 10.1084/jem.186.9.1487 PMC2199122

[26] Liu H, Jiang D (2011) Fractalkine/CX3CR1 and atherosclerosis. Clin Chim Acta 412: 1180–1186. 21492740 10.1016/j.cca.2011.03.036

[27] Hoppe AD, Swanson JA (2004) Cdc42, Rac1, and Rac2 display distinct patterns of activation during phagocytosis. Mol Biol Cell 15: 3509–3519. 15169870 10.1091/mbc.E03-11-0847 PMC491814

[28] Cervero P, Panzer L, Linder S (2013) Podosome reformation in macrophages: assays and analysis. Methods Mol Biol 1046: 97–121. 23868584 10.1007/978-1-62703-538-5_6

[29] Linder S, Nelson D, Weiss M, Aepfelbacher M (1999) Wiskott-Aldrich syndrome protein regulates podosomes in primary human macrophages. Proc Natl Acad Sci U S A 96: 9648–9653. 10449748 10.1073/pnas.96.17.9648 PMC22264

[30] Cammer M, Gevrey JC, Lorenz M, Dovas A, Condeelis J, et al (2009) The mechanism of CSF-1-induced Wiskott-Aldrich syndrome protein activation in vivo: a role for phosphatidylinositol 3-kinase and Cdc42. J Biol Chem 284: 23302–23311. 19561083 10.1074/jbc.M109.036384 PMC2749104

[31] Blundell MP, Bouma G, Metelo J, Worth A, Calle Y, et al (2009) Phosphorylation of WASp is a key regulator of activity and stability in vivo. Proc Natl Acad Sci U S A 106: 15738–15743. 19805221 10.1073/pnas.0904346106 PMC2736139

[32] Tzima E, Kiosses WB, del Pozo MA, Schwartz MA (2003) Localized cdc42 activation, detected using a novel assay, mediates microtubule organizing center positioning in endothelial cells in response to fluid shear stress. J Biol Chem 278: 31020–31023. 12754216 10.1074/jbc.M301179200

[33] Park H, Cox D (2011) Syk regulates multiple signaling pathways leading to CX3CL1 chemotaxis in macrophages. J Biol Chem 286: 14762–14769. 21388954 10.1074/jbc.M110.185181 PMC3083178

[34] Miskolci V, Spiering D, Cox D, Hodgson L (2013) A mix-and-measure assay for determining the activation status of endogenous Cdc42 in cytokine-stimulated macrophage cell lysates. Methods in Molecular Biology In Press. 10.1007/978-1-4939-0928-5_15 PMC4133094 24908304

[35] Rizzo MA, Springer GH, Granada B, Piston DW (2004) An improved cyan fluorescent protein variant useful for FRET. Nat Biotechnol 22: 445–449. 14990965 10.1038/nbt945

[36] Parrini MC, Lei M, Harrison SC, Mayer BJ (2002) Pak1 kinase homodimers are autoinhibited in trans and dissociated upon activation by Cdc42 and Rac1. Molecular Cell 9: 73–83. 11804587 10.1016/s1097-2765(01)00428-2

[37] Spiering D, Hodgson L (2011) Multiplex Imaging of Rho GTPase Activities in Living Cells In: Rivero F, editor. Methods in Molecular Biology. New York: Humana Press, Inc.

[38] Spiering D, Bravo-Cordero JJ, Moshfegh Y, Miskolci V, Hodgson L (2013) Quantitative Ratiometric Imaging of FRET-Biosensors in Living Cells. Methods Cell Biol 114: 593–609. 23931524 10.1016/B978-0-12-407761-4.00025-7 PMC3789067

[39] Spiering D, Hodgson L (2012) Multiplex Imaging of Rho Family GTPase Activities in Living Cells. Methods in Molecular Biology 827: 215–234. 22144278 10.1007/978-1-61779-442-1_15 PMC3241978

[40] Efron B, Tibshirani R (1993) An Introduction to the bootstrap. New York: Chapman & Hall. 436 p.

