# Supplementary figures and images for: A New Genetically Encoded Single-Chain Biosensor for Cdc42 Based on FRET, Useful for Live-Cell Imaging
Source: PLoS One. 2014 May 5;9(5):e96469. doi: 10.1371/journal.pone.0096469 (PMC4010534; doi:10.1371/journal.pone.0096469)

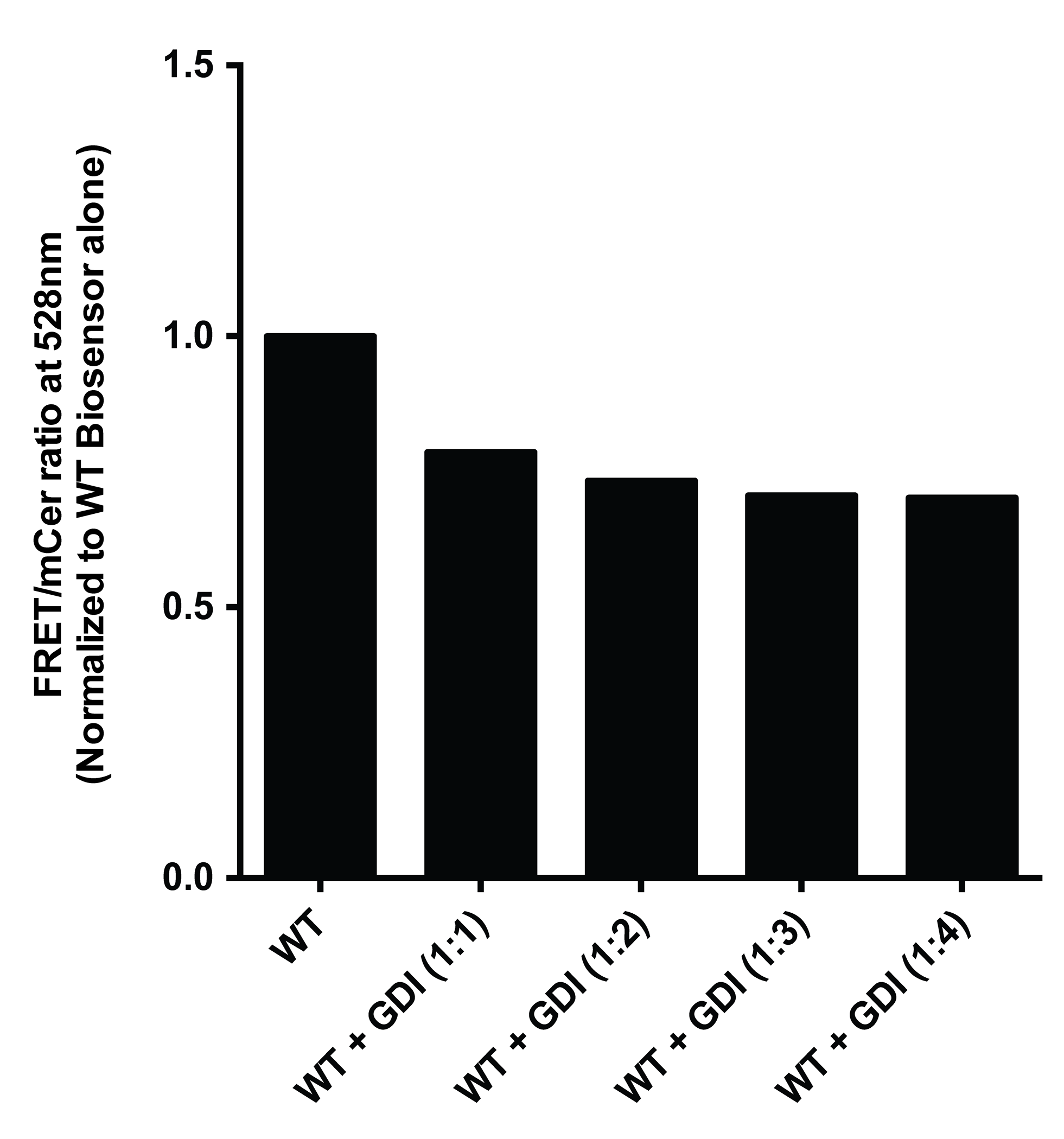

Supplement: Figure S1 — GDI titration effect on wild-type Cdc42 biosensor. Normalized FRET/mCer ratios of wild-type biosensor (WT) with and without co-expression with increasing concentrations of negative regulator (GDI) starting from 1∶1 ratio up to 4 folds excess GDI. (TIF) [file pone.0096469.s001.tif]

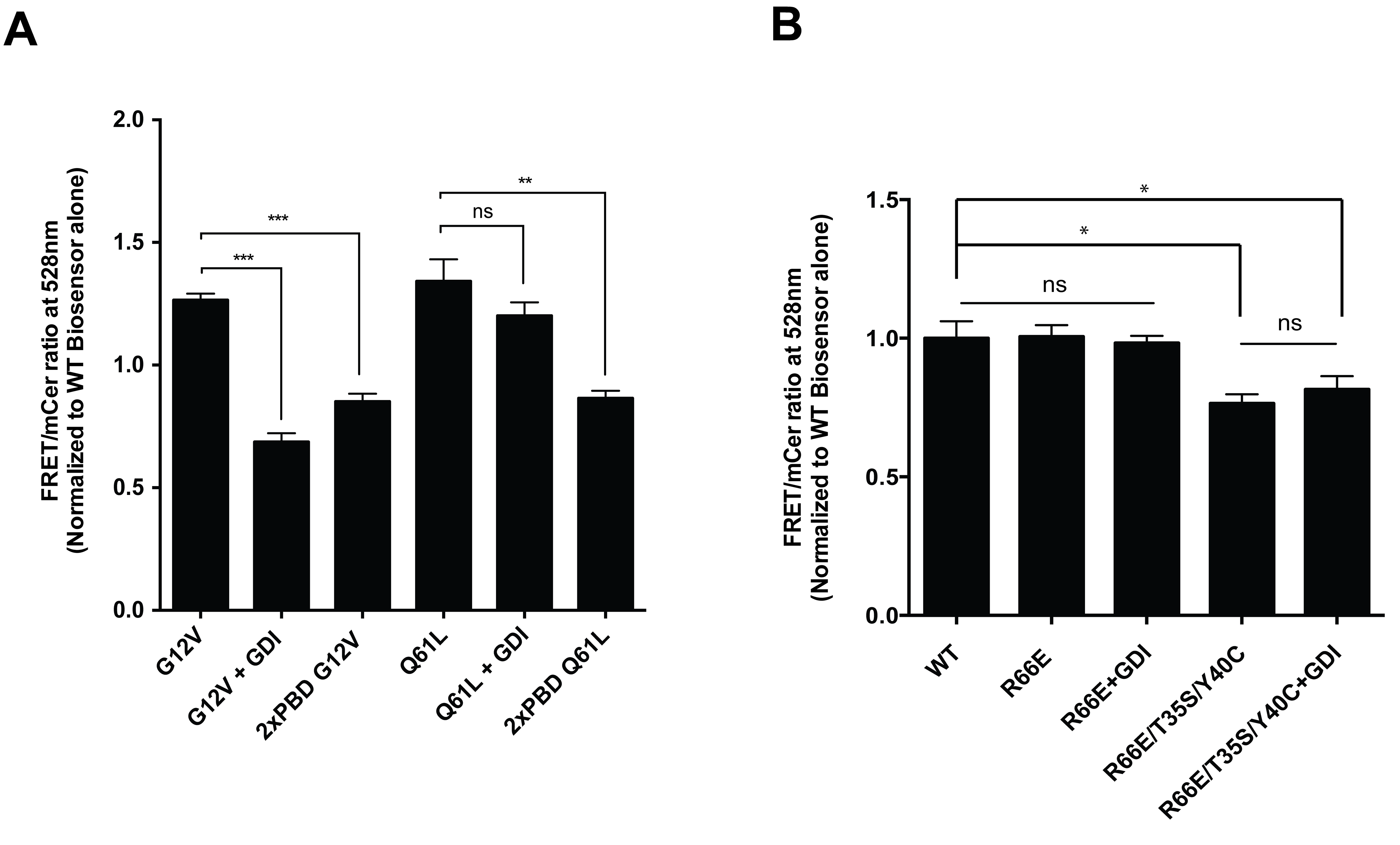

Supplement: Figure S2 — Characterization of GTPase-binding mutant of the Cdc42 biosensor. A) Normalized FRET/mCer ratios of wild-type (WT) and mutant forms of the biosensor containing a second GTPase-binding deficient mutation in PBD domain (2xPBD) with or without co-expression with negative regulator (GDI). **p<0.0062, ***p<0.0007, ns: non-significant. B) Normalized FRET/mCer ratios of wild-type (WT) and mutant forms of the biosensor containing a GDI-binding deficient mutation R66E with or without co-expression with GDI, as well as a combination of effector binding and GDI-binding mutations R66E/T35S/Y40C with or without co-expression with GDI. *<0.0112, ns: non-significant. (TIF) [file pone.0096469.s002.tif]

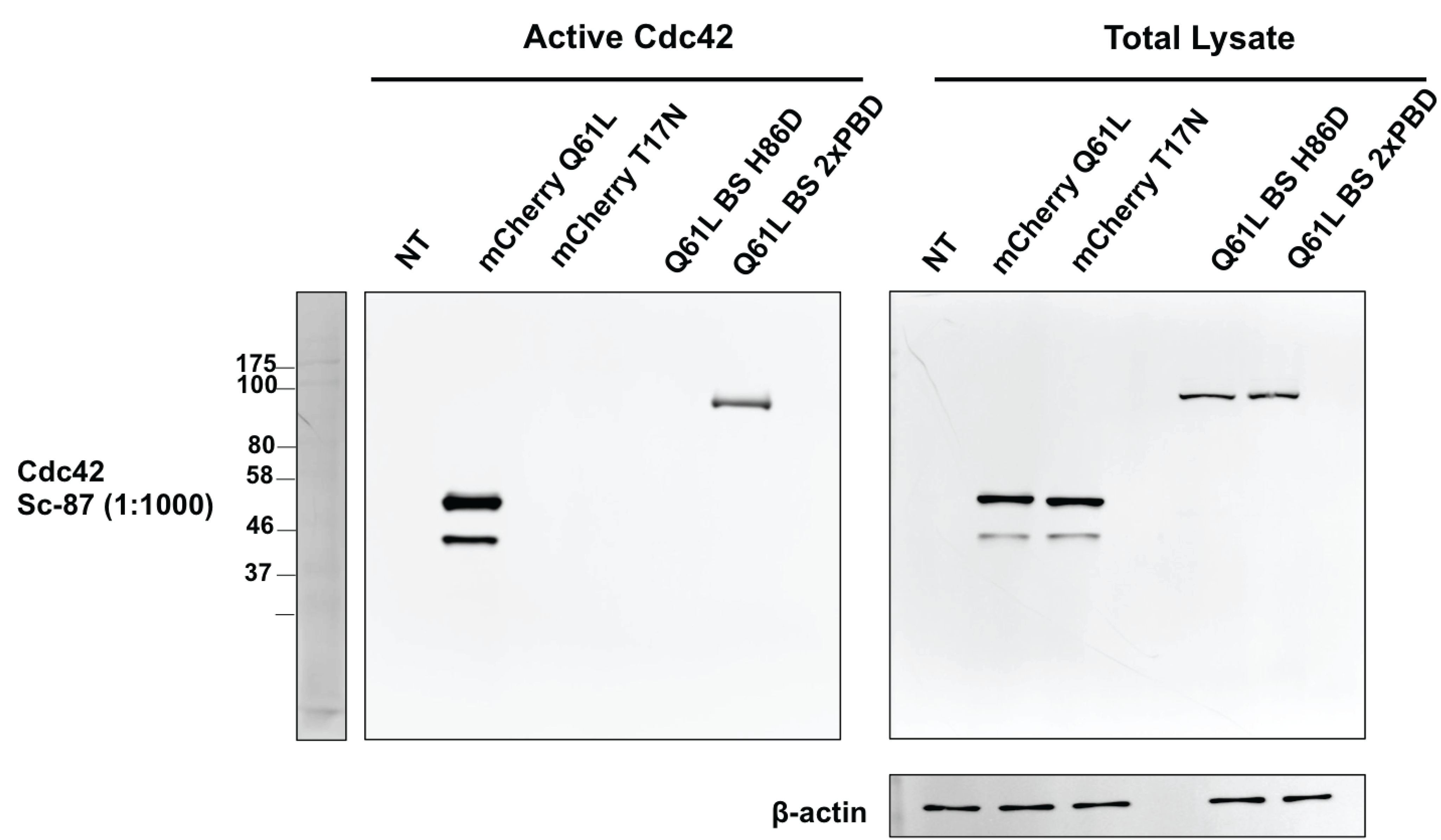

Supplement: Figure S3 — Cdc42 biosensor does not bind to endogenous effectors. Western blot of GST-PAK pulldown of active forms of mCherry tagged-Q61L constitutively active Cdc42 mutant, T17N dominant negative and Q61L constitutively active version of the biosensor with competent or non-binding mutations in the PBD1 domain (left). Total lysates and β-actin was used as a loading control (right). Here, we show that the biosensor with competent PBD cannot be pulled down using excess exogenous effector, showing that the built-in binding domain cannot be competed away by the downstream effectors thus minimizing the dominant negative effect. The biosensor can be pulled down only if both PBD domains contain the GTPase binding mutations (H83D/H86D). (TIF) [file pone.0096469.s003.tif]

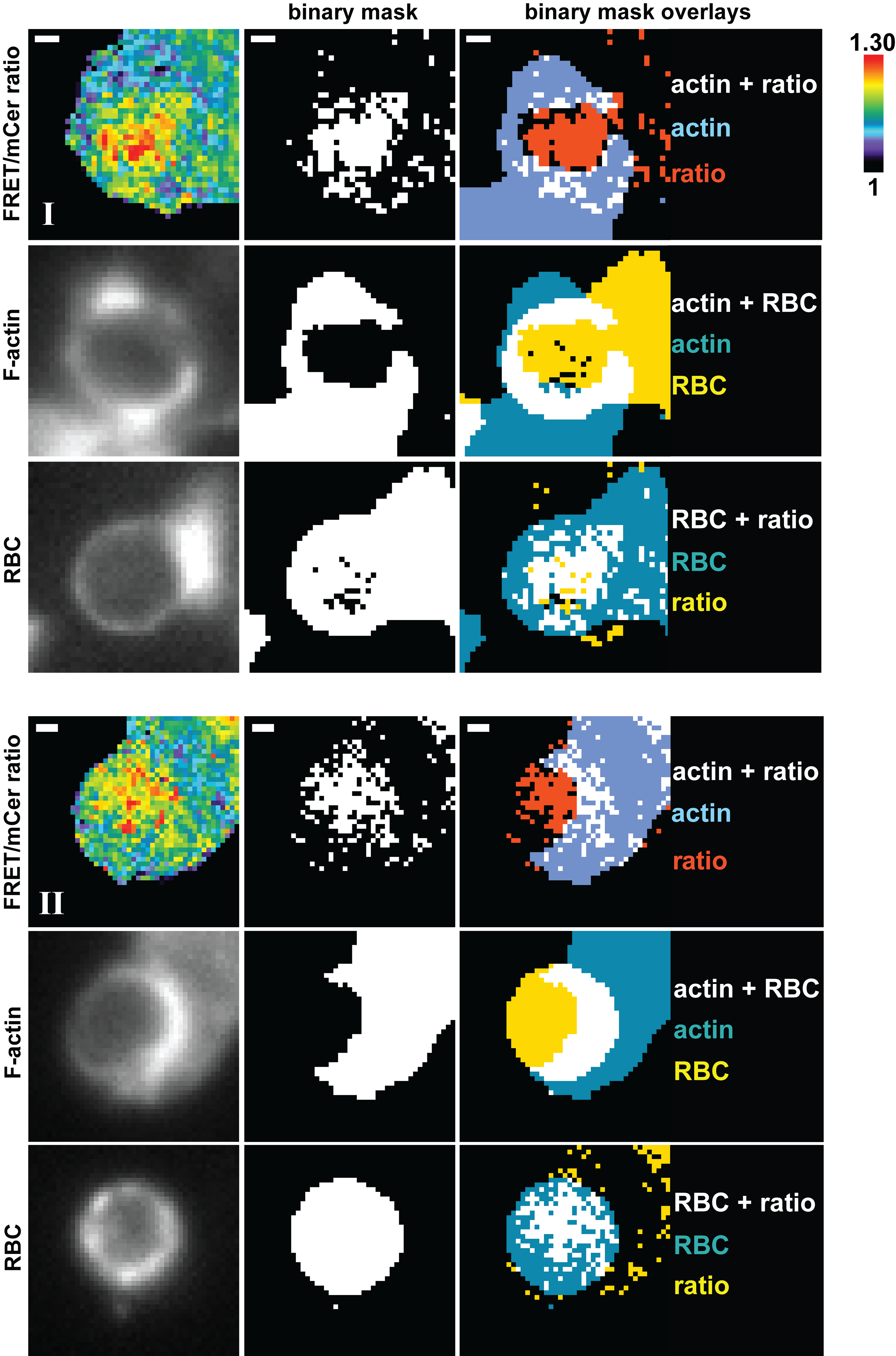

Supplement: Figure S4 — Relative localization of Cdc42 activity to F-actin at the phagosome. Zoomed view of phagosomes from image sets in Figure 4 are shown. Top and bottom panel sets correspond to cells I and II, indicated in the FRET/mCer ratio images. First column shows original images of FRET/mCer ratio, F-actin and RBC. Second column shows binary masks created for original images. In the third column, binary mask of F-actin was overlayed with that of FRET/mCer ratio to show the relative localization of Cdc42 activity to F-actin. Binary mask overlays of F-actin with RBC and RBC with FRET/mCer ratio are also shown. White scale bar = 1 µm. (TIF) [file pone.0096469.s004.tif]

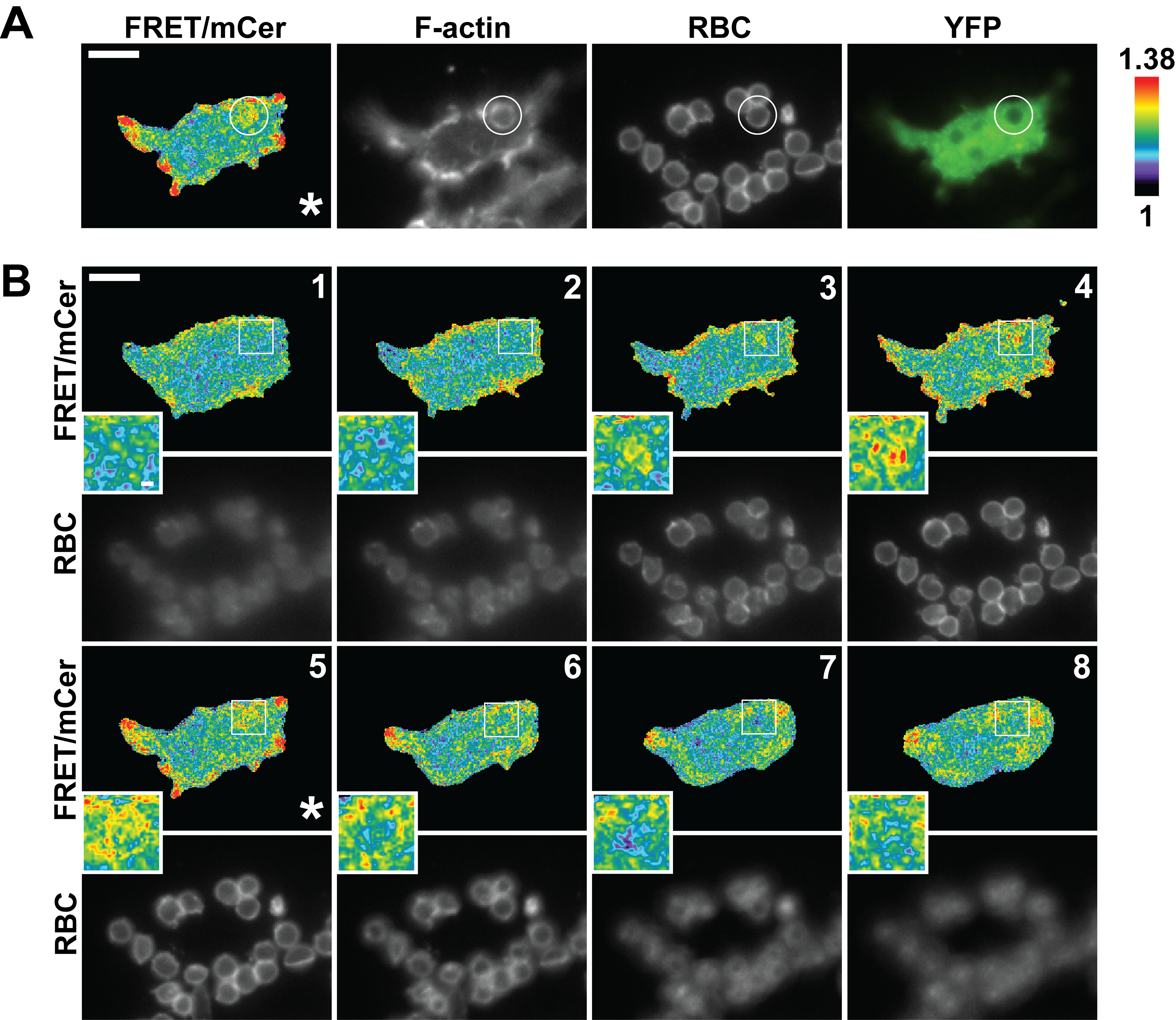

Supplement: Figure S5 — Ratiometric imaging of Cdc42 activity over serial planes of the phagosome. RAW/LR5 cells transiently expressing wt Cdc42 biosensor were imaged A) at optimal F-actin focal plane as in Figure 4C and B ) in Z-series at 1 µm-steps where the focal plane from A was set as the center (*). Planes 4-1 progress down towards the base of the phagocytic cup and below, while planes 6-8 move upwards from the F-actin plane. Shown is a representative image set of n = 6 cells. Scale bar for whole cell = 10 µm and zoomed inset = 1 µm. Imaged by oil/60X. (TIF) [file pone.0096469.s005.tif]

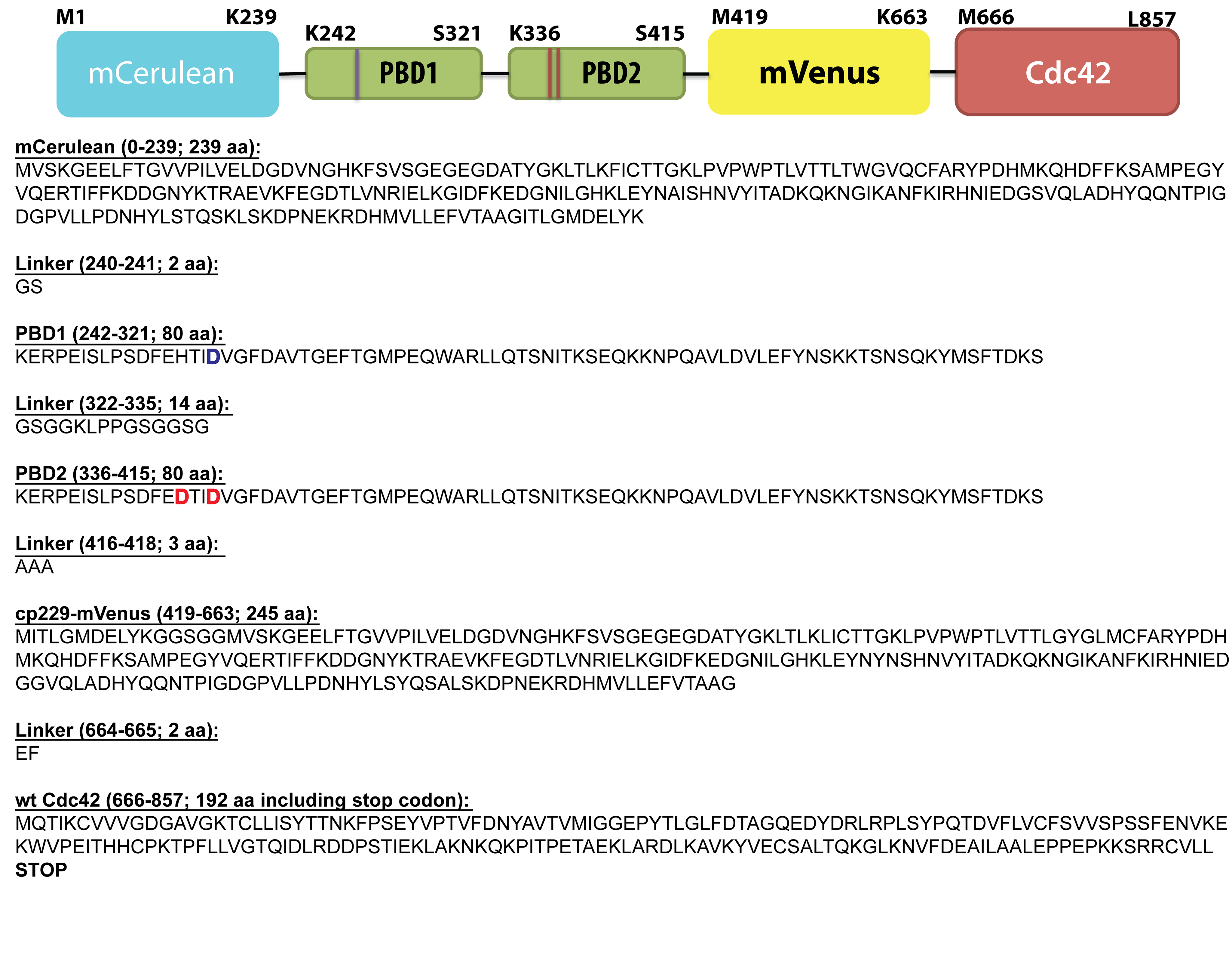

Supplement: Figure S6 — Amino acid sequence and the domain structure of the new biosensor for Cdc42. H86D mutation in the PBD1 is shown in blue, and H86/83D mutations in the PBD2 are shown in red. (TIF) [file pone.0096469.s006.tif]
